# C-753-02. Impact of Bleeding Disorders on Clinical Outcomes in Burn Patients: A Retrospective Cohort Study

**DOI:** 10.1093/jbcr/irag033.023

**Published:** 2026-04-06

**Authors:** Mare G Kaulakis, Catherine K Perez, Christopher J Fedor, Sarah M Tepe, Jenny A Ziembicki, Francesco M Egro

**Affiliations:** University of Pittsburgh Medical Center, Mercy Burn Center, Pittsburgh, PA; University of Pittsburgh Medical Center, Mercy Burn Center, Pittsburgh, PA; University of Pittsburgh Medical Center Department of Plastic Surgery, Pittsburgh, PA; University of Pittsburgh Medical Center, Pittsburgh, PA; University of Pittsburgh Medical Center, Pittsburgh, PA; University of Pittsburgh Medical Center, Pittsburgh, PA

## Abstract

**Introduction:**

Burn-induced coagulopathy is a common complication of burn injuries associated with increased morbidity and mortality. However, there is a lack of studies evaluating patients with pre-existing bleeding disorders as distinct from those who develop secondary coagulopathy. This study aims to assess outcomes in burn patients with pre-existing disorders versus those who develop coagulopathy.

**Methods:**

A retrospective analysis was conducted using data from the Burn Care Quality Platform (BCQP) from 2013 to 2022. Demographics, burn characteristics, comorbidities, and hospitalization outcomes were analyzed using paired t-tests, chi-squared tests, and multivariable logistic regression models.

**Results:**

Of 286 478 burn patients, 2825 (1.0%) had pre-existing bleeding disorders, and 106 (0.04%) had burn-induced coagulopathies (including DIC). Patients with bleeding disorders were significantly older (62.5 vs. 37.0 years; *p*<.001), and had similar TBSA (7.5% vs. 7.48%; *p*=.94). In contrast, patients with a coagulopathy had significantly larger burns (39.3% vs. 7.6%; *p*<.001) and were also older (47.9 vs. 37.0; *p*<.001). Patients with DIC were also found to be older (46.3 vs. 37.0; *p*=.0095) and had larger burns (50.8% vs 7.6%; *p*<.001).

Bleeding disorders were linked to higher mortality in unadjusted models (OR = 2.36; *p*<.001), but not after adjustment for age, TBSA, and sex (aOR = 1.05; *p*=.67). In a combined model including all three bleeding disorder variables, coagulopathy (aOR = 6.91; *p*<.001) and DIC (aOR = 24.37; *p*<.001) were independently associated with mortality.

Bleeding disorders were associated with increased hospital length of stay (β = 0.95; *p*=.003), but not with increased odds of skin graft loss, septicemia, or severe sepsis/shock. Coagulopathies were associated with a significantly longer hospital stay (β = 13.05; *p*<.001) and increased odds of non-infectious skin graft loss (aOR = 8.25; *p*<.001) and septicemia (aOR = 73.48; *p*<.001). DIC was associated with an increased odds of septicemia (aOR = 12.28; *p*<.001).

**Conclusions:**

These findings highlight the importance of early recognition and aggressive management of coagulopathies in burn patients, while suggesting standard treatment protocols can generally be maintained for those with chronic bleeding disorders.

**Applicability of Research to Practice:**

Our results suggest pre-existing bleeding disorders may not warrant separate treatment pathways, which could inform future clinical trial inclusion criteria and enhance the generalizability of burn research.

**Funding for the Study:**

N/A.

